# Hepatitis B virus pregenomic RNA is associated with malignant phenotypes and postoperative survival in HBV-related intrahepatic cholangiocarcinoma

**DOI:** 10.1186/s13027-026-00792-1

**Published:** 2026-09-10

**Authors:** Qiyu Tian, Wen Li, Pengpeng Li, Yuanyuan Wang, Judian Yu, Jian Yu, Gang Huang

**Affiliations:** 1 The Department of General Surgery, Eastern Hepatobiliary Surgery Hospital, Naval Medical University, 225 Changhai Road, Shanghai, 200438 China; 2 The Third Department of Hepatic Surgery, Eastern Hepatobiliary Surgery Hospital, Naval Medical University, Shanghai, China

**Keywords:** Intrahepatic cholangiocarcinoma, Hepatitis B virus, Pregenomic RNA, Infection-associated cancer, Tumor stemness, Postoperative survival

## Abstract

**Background:**

Chronic hepatitis B virus (HBV) infection contributes to infection-associated liver carcinogenesis. However, the biological and clinical relevance of HBV pregenomic RNA (pgRNA), a marker of residual viral transcriptional activity, in HBV-related intrahepatic cholangiocarcinoma (iCCA) remains insufficiently defined.

**Methods:**

This single-center retrospective exploratory translational study included 45 patients with HBV-related iCCA who underwent curative resection after long-term nucleos(t)ide analogue therapy and had suppressed preoperative serum HBV DNA. Tissue pgRNA expression was measured by quantitative reverse-transcription polymerase chain reaction (qRT-PCR), serum pgRNA was quantified by simultaneous amplification and testing (SAT), and spatial localization was assessed by immunofluorescence–fluorescence in situ hybridization (IF-FISH). Stable pgRNA-overexpressing HuCCT1 and RBE models were used to evaluate cell viability, migration, invasion, colony formation and stemness-related protein expression. Associations between serum pgRNA status and postoperative outcomes were examined using Kaplan–Meier, Cox regression and restricted mean survival time analyses.

**Results:**

Tumor tissues showed lower overall pgRNA expression than paired adjacent non-tumor liver tissues, whereas pgRNA signals were detectable in a subset of CK19-positive tumor epithelial cells. In HuCCT1 and RBE cells, pgRNA overexpression was associated with increased cell viability, migration, invasion, colony formation and upregulation of multiple stemness-related proteins. In the clinical analysis, the serum pgRNA HIGH group had poorer overall survival (adjusted HR = 5.22, 95% CI 1.82–14.95; *P* = 0.002) and recurrence-free survival (adjusted HR = 3.51, 95% CI 1.26–9.79; *P* = 0.017) than the BLQ group after adjustment for tumor size.

**Conclusions:**

HBV pgRNA may be linked to malignant and stemness-related phenotypes in HBV-related iCCA. High preoperative serum pgRNA was associated with poorer postoperative overall and recurrence-free survival in this exploratory cohort. These findings support validation of pgRNA as a candidate infection-related biomarker in larger prospective multicenter studies.

**Supplementary Information:**

The online version contains supplementary material available at https://doi.org/10.1186/s13027-026-00792-1.

## Introduction

Intrahepatic cholangiocarcinoma (iCCA) is a primary liver malignancy arising from intrahepatic biliary epithelium and represents the second most common primary hepatic cancer after hepatocellular carcinoma [1]. iCCA is clinically aggressive and biologically heterogeneous, with substantial variation in molecular features [2, 3], etiological backgrounds [4] and geographic distribution [3]. In regions where hepatitis B virus (HBV) infection is endemic, chronic HBV infection has been recognized as a potential contributor to iCCA development [5, 6] and may define a biologically distinct infection-associated tumor context [7].

Epidemiological and genomic evidence supports a link between HBV infection and iCCA. HBV infection has been associated with an increased risk of cholangiocarcinoma [5, 6], and viral integration events have been detected in HBV-related iCCA tumor and adjacent non-tumor tissues [8]. These observations support the hypothesis that residual viral transcription, viral integration and HBV-associated genomic instability may contribute to cholangiocarcinogenesis; however, the relationship between residual HBV transcriptional signals and the biological behavior of iCCA remains incompletely characterized.

HBV pregenomic RNA (pgRNA) is a 3.5-kb transcript that serves as the template for reverse transcription during the HBV life cycle and is classically transcribed from covalently closed circular DNA (cccDNA). During long-term nucleos(t)ide analogue (NA) therapy, serum HBV DNA may be suppressed whereas residual viral transcriptional activity can persist. Circulating HBV RNA/pgRNA has therefore emerged as a candidate marker of ongoing HBV transcription [9, 10], although its interpretation in tumor-bearing patients may be affected by cccDNA activity, integrated HBV sequences, tumor burden, antiviral treatment and host factors [11].

Studies in HBV-related hepatocellular carcinoma have suggested that pgRNA may be associated with stemness-related phenotypes and clinical outcomes [12, 13]. Mechanistically, reciprocal regulation between pgRNA and RNA-binding protein IGF2BP3 has been reported [14]. iCCA also contains stemness-related cellular states characterized by markers such as CD44, ALDH1A1, CK19 and CD24 [15, 16] and by signaling pathways including Wnt/β-catenin [15] and Hippo/YAP [17]. These observations led us to hypothesize that HBV pgRNA may be linked to malignant and stemness-related phenotypes in HBV-related iCCA.

In this study, we performed an exploratory translational analysis of HBV pgRNA in HBV-related iCCA by integrating tissue localization, cell-based functional assays and postoperative outcome data. Specifically, we aimed to characterize the tissue distribution and epithelial localization of pgRNA, evaluate whether pgRNA overexpression is associated with malignant and stemness-related phenotypes in iCCA cells, and examine the exploratory association between preoperative serum pgRNA status and postoperative outcomes.

## Materials and methods

### Study design, patients and specimens

This was a single-center retrospective exploratory translational study reported in accordance with the STROBE statement and with attention to prognostic biomarker reporting principles [18, 19]. Patients with iCCA who underwent radical resection at the Eastern Hepatobiliary Surgery Hospital, Naval Medical University, between July 2011 and August 2020 were screened. The study was approved by the Ethics Committee of Eastern Hepatobiliary Surgery Hospital, Naval Medical University, and written informed consent was obtained from all patients before surgery.

The inclusion criteria were as follows: (1) pathologically confirmed iCCA after surgery; (2) HBsAg positivity for at least 6 months before surgery; (3) long-term treatment with NAs for at least 12 months and serum HBV DNA < 20 IU/mL; and (4) radical surgical resection (R0 resection).

The exclusion criteria were: (1) coinfection with hepatitis C virus, hepatitis D virus or human immunodeficiency virus; (2) autoimmune hepatitis, primary biliary cholangitis or other autoimmune liver diseases; (3) alcoholic liver disease, defined as alcohol intake > 40 g/day in men or > 20 g/day in women for more than 5 years; (4) concurrent hepatocellular carcinoma or other malignancies; (5) decompensated cirrhosis (Child–Pugh class B/C) or liver failure; (6) receipt of any preoperative antitumor treatment, including chemotherapy, radiotherapy or interventional therapy; and (7) incomplete clinical or follow-up data.

Because HBV-related iCCA with long-term NA therapy and suppressed preoperative HBV DNA is uncommon, no formal power calculation was performed. The sample size was determined by the number of eligible patients with available serum, paired tissue specimens and follow-up data during the study period. Baseline demographic characteristics, including age and sex, and clinicopathological data, including tumor burden, TNM/AJCC stage and serum tumor markers, were collected.

The primary clinical endpoints were OS and RFS. OS was defined as the time from surgery to death from any cause. RFS was defined as the time from surgery to the first occurrence of documented tumor recurrence or death from any cause, whichever occurred first. Recurrence included local recurrence, regional lymph node metastasis, or distant metastasis and was assessed according to postoperative imaging and clinical records, with pathological confirmation when available. Patients who remained alive and recurrence-free were censored at the date of the last valid follow-up confirming absence of recurrence. For OS, patients who remained alive were censored at the date they were last known to be alive. The median follow-up time was approximately 24 months, and the last follow-up date was December 2025.

Peripheral blood (10 mL) was collected after overnight fasting before surgery. Samples were allowed to stand at room temperature for 30 min and were then centrifuged at 3000 rpm for 10 min at 4 °C. Serum was separated, aliquoted and stored at − 80 °C. Tumor tissues and paired adjacent non-tumor liver tissues (> 1 cm from the tumor margin) were collected during surgery. Part of each specimen was snap frozen in liquid nitrogen and stored at − 80 °C, and the remaining tissue was fixed in 4% paraformaldehyde and embedded in paraffin. Fresh tumor specimens were pathologically confirmed to contain > 80% tumor cells, whereas adjacent non-tumor tissues were confirmed to be free of tumor infiltration.

### Tissue and serum pgRNA assays

#### Tissue pgRNA quantification

Total RNA was extracted from tissue samples using TRIzol reagent and reverse transcribed into cDNA. pgRNA expression was measured by SYBR Green-based qRT-PCR, with GAPDH used as the internal control. The pgRNA primer sequences were as follows: forward, 5′-TGTGGAGTTACTCTCGTTTTTGC-3′; reverse, 5′-AAGGCTTCCCGATACAGAGC-3′. Relative pgRNA expression was calculated using the comparative Ct (2^−ΔCt) method after normalization to GAPDH [20]. Primer sequences and internal-control normalization are reported to facilitate reproducibility, consistent with MIQE reporting principles [21].

#### Serum pgRNA absolute quantification

Serum pgRNA testing was performed by Shanghai Rendu Biotechnology Co., Ltd. (Shanghai, China) using simultaneous amplification and testing (SAT). In brief, HBV RNA was extracted from serum using specific capture probes, amplified isothermally by M-MLV reverse transcriptase and T7 RNA polymerase, and detected in real time using molecular beacon probes targeting pgRNA-specific sequences. Testing was performed on an AutoSAT fully automated nucleic acid detection system. The lower limit of detection was 50 copies/mL, and the linear quantification range was 10^2–10^8 copies/mL. Positive calibrators, negative controls and internal controls were included in each batch for quality control. For exploratory clinical stratification, values < 100 copies/mL were defined as below the lower limit of quantification (BLQ), and quantifiable samples were divided into LOW and HIGH groups using the median value among quantifiable patients. This cutoff was data-driven and exploratory rather than a clinically validated threshold.

#### Immunofluorescence–fluorescence in situ hybridization

IF-FISH was performed by Wuhan Servicebio Technology Co., Ltd. Tumor tissue paraffin sections (4 μm thick) from five randomly selected patients were used to detect the spatial relationship between pgRNA and CK19. Image acquisition was performed under identical settings for comparable regions. Colocalization analyses were conducted in Fiji using the Coloc2 workflow [22, 23]. Pearson’s correlation coefficients, Costes automatic thresholding and Manders’ coefficients were used to assess signal overlap and reduce background-driven colocalization bias [24, 25]. Because dedicated RNase-treated or scramble-probe controls were not available for these archival sections, the IF-FISH results were interpreted as descriptive spatial evidence rather than definitive proof of the intracellular origin of pgRNA.

### Cell lines, vector construction and functional assays

#### Cell culture

The human iCCA cell lines HuCCT1 and RBE were used. Both cell lines were purchased from Shanghai Kuisai Biotechnology Co., Ltd. and authenticated by short tandem repeat profiling; mycoplasma testing was negative for all cell lines. HuCCT1 and RBE cells were cultured in RPMI-1640 medium (Gibco, USA) supplemented with 10% fetal bovine serum (Gibco, USA), 100 U/mL penicillin and 100 µg/mL streptomycin at 37 °C in a humidified incubator containing 5% CO₂. Logarithmically growing cells were used for experiments. The parental HuCCT1 cells were negative for HBV DNA, pgRNA and HBsAg by laboratory testing.

#### Vector construction and stable transfection

HuCCT1 and RBE cells were transfected with a pgRNA-expression plasmid carrying a puromycin resistance gene or the pcDNA3.1(+) empty vector using Lipofectamine 3000 (Invitrogen). The construct was designed to express HBV pgRNA in iCCA cells and was not intended to reconstitute a complete HBV replication system. After 48 h, the medium was replaced with selection medium containing puromycin (2 µg/mL), and stable cell lines were established after 14 days of selection. Single clones were obtained by limiting dilution. qRT-PCR confirmed marked and stable pgRNA overexpression in both cell models. HBV-related protein and antigen assays were performed to assess whether the system generated a broader HBV protein expression profile.

#### Protein expression validation

For western blotting, total cellular protein was extracted using RIPA lysis buffer containing 1% protease and phosphatase inhibitors, and protein concentrations were measured using a BCA assay. Proteins (20 µg) were separated by 4–12% SDS-PAGE and transferred to PVDF membranes. Membranes were blocked with rapid blocking buffer for 30 min at room temperature and incubated with primary antibodies overnight at 4 °C. After washing three times with PBST for 5 min each, membranes were incubated with fluorescent secondary antibodies at 4 °C for 1 h. HBcAg, HBx, CD44, ALDH1A1, CK19 and CD24 were assessed, with β-actin used as the loading control. HepG2.2.15 cells were used as a positive control for HBV-associated proteins.

For ELISA, cell culture supernatants were collected and analyzed using the Human HBsAg ELISA Kit (ELK Biotechnology, ELK9601; detection range 0.31–20 ng/mL; sensitivity 0.19 ng/mL) and the Human HBeAg ELISA Kit (Abbkine, KTE63071; detection range 6.25–100 IU/L; minimum detectable dose < 0.5 IU/L) according to the manufacturers’ instructions. Values below the respective assay detection limits were reported as not detected (ND).

#### Cell functional assays

All functional assays were performed in three independent biological replicates.

For the CCK-8 cell-viability assay, HuCCT1 and RBE cells were seeded into 96-well plates at 3000 cells per well. At 0, 24, 48 and 72 h, 10 µL of CCK-8 reagent was added to each well, followed by incubation at 37 °C for 1 h. Absorbance at 450 nm was measured using a microplate reader.

For the wound-healing assay, cells were seeded into 6-well plates and grown to > 90% confluence. A linear scratch was created using a 200-µL pipette tip. Detached cells were removed by PBS washing, and cells were incubated in serum-free medium containing mitomycin C (1 µg/mL) for 1 h to inhibit proliferation. After two PBS washes, fresh serum-free medium was added, and images were captured at 0 and 48 h under an inverted microscope (40×). Wound areas were measured using Fiji, and wound closure rates were calculated.

For Transwell migration and invasion assays, 24-well Transwell chambers with 8-µm pore polycarbonate membranes were used. For migration assays, 5 × 10⁴ cells were seeded into the upper chamber in serum-free medium, and complete medium containing 10% FBS was added to the lower chamber. Cells were incubated for 24 h. For invasion assays, the upper chamber was precoated with 50 µL of Corning Matrigel diluted 1:8 and allowed to solidify, after which 1 × 10⁵ cells were seeded into the upper chamber and incubated for 48 h. At the end of the experiment, cells remaining on the upper surface were removed, and cells that migrated or invaded through the membrane were fixed with 4% paraformaldehyde for 30 min, stained with 0.1% crystal violet for 20 min and washed with PBS. After air drying, five random fields (20×) were imaged under a fluorescence microscope, and cells were counted for analysis.

For the colony-formation assay, cells from the control and pgRNA-overexpression groups were seeded into 6-well plates at 500 cells per well and cultured in medium containing 10% fetal bovine serum for 14 days. After fixation and staining, colonies larger than 1 mm in diameter were counted. Three replicate wells were used per group, and results are reported as the mean number of colonies.

### Statistical analysis

Categorical variables are presented as frequencies and percentages, and continuous variables as mean ± standard deviation or median (interquartile range), as appropriate. Baseline continuous variables were compared among serum pgRNA groups using the Kruskal–Wallis test, and categorical variables were compared using Fisher’s exact test, with Monte Carlo simulation (200,000 replicates) for larger contingency tables. Paired tumor and adjacent non-tumor tissue pgRNA expression was compared after log₁₀ transformation using a paired t-test. Tumor or adjacent non-tumor tissue pgRNA expression across serum pgRNA groups was compared on Ct-normalized values using Welch’s one-way ANOVA, and correlations between quantifiable serum pgRNA and tissue pgRNA were assessed using Spearman’s rank correlation. Cell-based experiments were performed in three independent biological replicates and analyzed according to the experimental design. All statistical tests were two-sided.

Survival curves were generated using the Kaplan–Meier method, and between-group differences were compared using the log-rank test. Cox proportional hazards regression was used to estimate hazard ratios (HRs) and 95% confidence intervals (CIs). Given the limited cohort size, a parsimonious adjustment strategy was used. The primary multivariable models for both OS and RFS included serum pgRNA group and tumor size. Sensitivity analyses used alternative pgRNA parameterizations and additional adjustment for AJCC stage and/or log₂-transformed CA19-9. The proportional hazards assumption was assessed using Schoenfeld residuals [26].

Restricted mean survival time (RMST) analyses at 18 and 24 months were used to quantify absolute survival differences [27], with Holm adjustment for the three pairwise comparisons within each endpoint and truncation time. Associations of tissue pgRNA expression with OS and RFS were explored using Cox models with HRs expressed per two-fold increase and adjustment for tumor size. When the proportional hazards assumption was violated for tumor-tissue pgRNA, time-varying coefficient models and a 24-month piecewise Cox sensitivity analysis were used. Statistical analyses were performed using R software (version 4.5.2), and *P* < 0.05 was considered statistically significant.

## Results

### Tissue localization, functional relevance and clinical association of HBV pgRNA

#### Cohort characteristics and exploratory serum pgRNA stratification

Among the 45 patients with HBV-related iCCA included in the clinical analysis, all had suppressed preoperative serum HBV DNA after long-term antiviral therapy (Supplementary Fig. S1). Serum HBV pgRNA < 100 copies/mL was defined as BLQ. Quantifiable samples were divided using the median value of 2700 copies/mL into LOW and HIGH groups, yielding BLQ (< 100 copies/mL, *n* = 30), LOW (100–2700 copies/mL, *n* = 8) and HIGH (> 2700 copies/mL, *n* = 7). Baseline characteristics are shown in Table 1. Tumor size differed among groups (*P* = 0.008), with larger tumors in the HIGH group, whereas CA19-9 did not differ significantly (*P* = 0.506). Tumor size was therefore included in the primary OS and RFS models.

**Table 1:** Baseline characteristics by serum pgRNA group

Characteristics	Total (*n* = 45)	BLQ (*n* = 30)	LOW (*n* = 8)	HIGH (*n* = 7)	*P* value
Demographic characteristics					
Age, years	58.0 (53.0–63.0)	58.5 (53.2–64.8)	55.5 (52.0–58.8)	59.0 (55.5–62.0)	0.467
Sex, n (%)					1.000
Male	35 (77.8)	23 (76.7)	6 (75.0)	6 (85.7)	
Female	10 (22.2)	7 (23.3)	2 (25.0)	1 (14.3)	
Tumor characteristics					
Tumor size, cm	5.9 (4.2–10.0)	5.0 (4.0–7.4)	5.5 (4.6–9.7)	10.7 (10.3–11.8)	0.008
Tumor number, n (%)					0.091
Solitary	30 (66.7)	22 (73.3)	6 (75.0)	2 (28.6)	
Multiple (≥ 2)	15 (33.3)	8 (26.7)	2 (25.0)	5 (71.4)	
T category, n (%)					0.320
T1	21 (46.7)	15 (50.0)	5 (62.5)	1 (14.3)	
T2	11 (24.4)	8 (26.7)	1 (12.5)	2 (28.6)	
T3	8 (17.8)	4 (13.3)	2 (25.0)	2 (28.6)	
T4	5 (11.1)	3 (10.0)	0 (0.0)	2 (28.6)	
N category, n (%)					0.672
N0	39 (86.7)	25 (83.3)	8 (100.0)	6 (85.7)	
N1	6 (13.3)	5 (16.7)	0 (0.0)	1 (14.3)	
AJCC 8th edition stage, n (%)					0.467
I	19 (42.2)	13 (43.3)	5 (62.5)	1 (14.3)	
II	9 (20.0)	6 (20.0)	1 (12.5)	2 (28.6)	
III	17 (37.8)	11 (36.7)	2 (25.0)	4 (57.1)	
Serological markers					
AFP, ng/mL	3.6 (2.5–6.9)	3.6 (2.5–6.8)	3.0 (2.8–6.2)	3.8 (2.3–6.9)	0.970
CA19-9, U/mL	46.2 (21.8–305.3)	44.3 (19.6–166.6)	134.0 (7.2–329.8)	94.2 (38.0–554.4)	0.506

Abbreviations: pgRNA, pregenomic RNA; BLQ, below the lower limit of quantification; AJCC, American Joint Committee on Cancer; AFP, alpha-fetoprotein; CA19-9, carbohydrate antigen 19 − 9; IQR, interquartile range

Notes: Continuous variables are presented as median (IQR), and categorical variables as n (%). Patients were classified according to preoperative serum pgRNA levels as BLQ (< 100 copies/mL), LOW (100–2700 copies/mL), or HIGH (> 2700 copies/mL). P values represent overall comparisons among the three groups. Continuous variables were compared using the Kruskal–Wallis test. Categorical variables were compared using Fisher’s exact test, with Monte Carlo simulation (200,000 replicates) for larger contingency tables. AFP and CA19-9 values reported with a greater-than sign were assigned the corresponding reporting-limit value for statistical analysis. All patients were classified as M0. A two-sided *P* value < 0.05 was considered statistically significant

#### Spatial colocalization of pgRNA and CK19 in tumor tissues

**Fig. 1:**
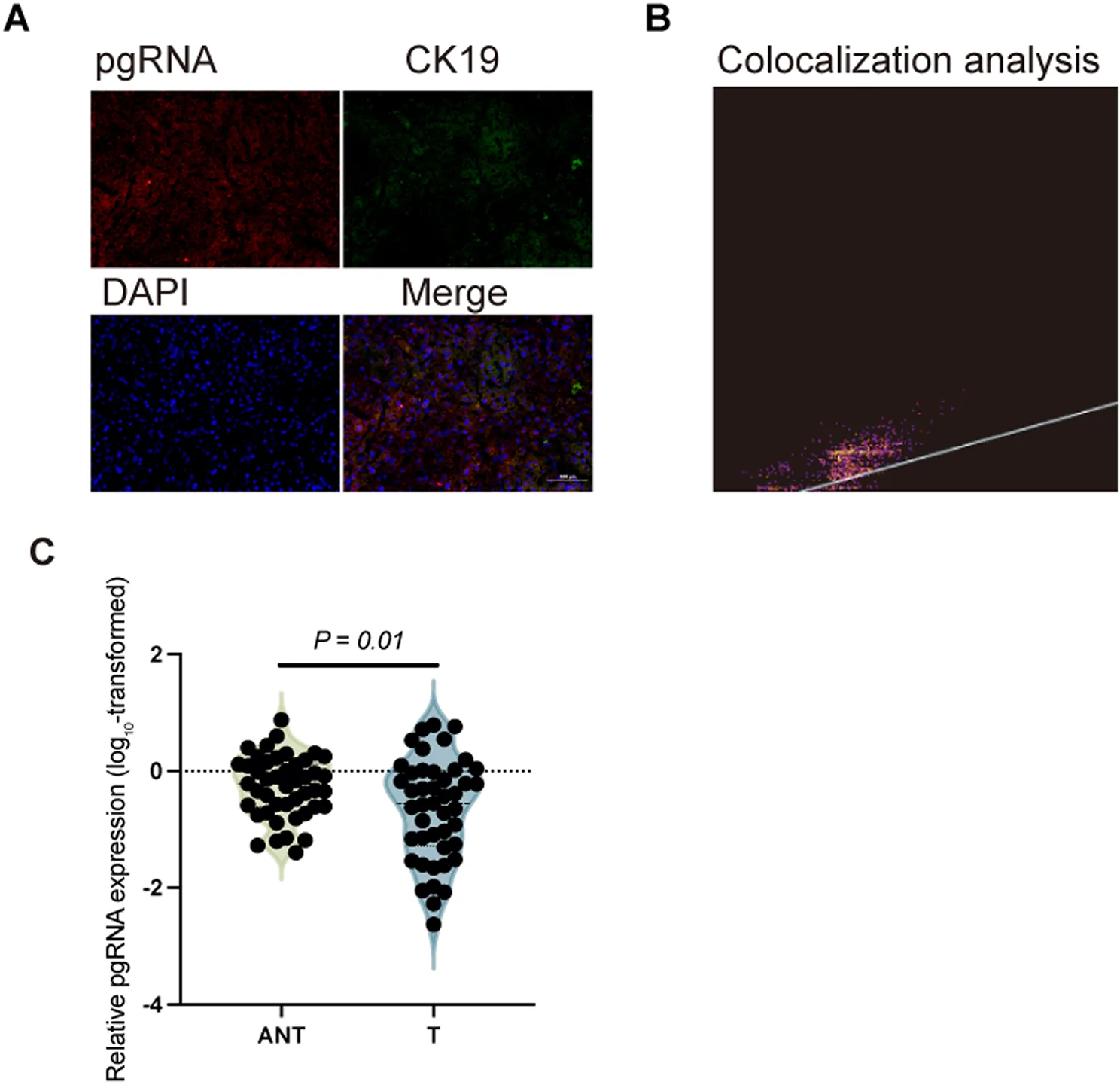
Detection and expression of pgRNA in intrahepatic cholangiocarcinoma tissues. (**A**) Representative fluorescence images showing pgRNA, CK19, DAPI, and merged signals in iCCA tissue. (**B**) Colocalization analysis of pgRNA and CK19 signals. (**C**) Comparison of relative pgRNA expression between paired adjacent non-tumor tissues (ANT) and tumor tissues (T) from 45 patients. Individual data points are shown, and the exact *P* value is indicated. Scale bar is shown in the image

IF–FISH showed pgRNA signals (red) in both nuclear and cytoplasmic compartments. In CK19-positive (green) biliary epithelial regions, pgRNA signals showed spatial colocalization with CK19 (Fig. 1A, B). Pixel-level colocalization analysis using the Fiji Coloc2 plugin showed a moderate positive correlation between the two signals (Pearson’s *r* = 0.64). After Costes automatic thresholding, the thresholded Pearson’s *r* was 0.56; thresholded Manders’ coefficients were *t*M1 = 0.720 and *t*M2 = 0.892. Costes randomization testing supported overlap beyond that expected by chance. The five IF–FISH cases included BLQ, LOW and HIGH serum pgRNA strata (Supplementary Table S2). Given the absence of RNase-treated and scramble-probe controls, these findings were interpreted as descriptive spatial evidence rather than proof of the intracellular origin of pgRNA.

#### Comparison of pgRNA expression between tumor and adjacent non-tumor liver tissues

pgRNA expression in paired tumor and adjacent non-tumor liver tissues was measured by qRT-PCR and expressed as relative pgRNA abundance. After log₁₀ transformation, paired t-test analysis showed significantly lower pgRNA expression in tumor tissues than in paired adjacent non-tumor liver tissues (*P* = 0.010; Fig. 1C).

Across serum pgRNA strata, neither tumor-tissue pgRNA expression (Welch’s ANOVA *P* = 0.574) nor adjacent non-tumor tissue pgRNA expression (*P* = 0.093) differed significantly among BLQ, LOW and HIGH groups (Supplementary Fig. S2A, B). Among the 15 patients with quantifiable serum pgRNA, serum pgRNA was not significantly correlated with tumor-tissue pgRNA (Spearman ρ = 0.314; *P* = 0.254) but showed a moderate positive correlation with adjacent non-tumor tissue pgRNA (ρ = 0.532; *P* = 0.044; Supplementary Fig. S2C, D). These correlations were considered exploratory because of the small quantifiable subset.

### pgRNA overexpression is associated with enhanced malignant phenotypes and stemness-related features in iCCA cells

#### Establishment and validation of a stable pgRNA-overexpression cell model

**Fig. 2:**
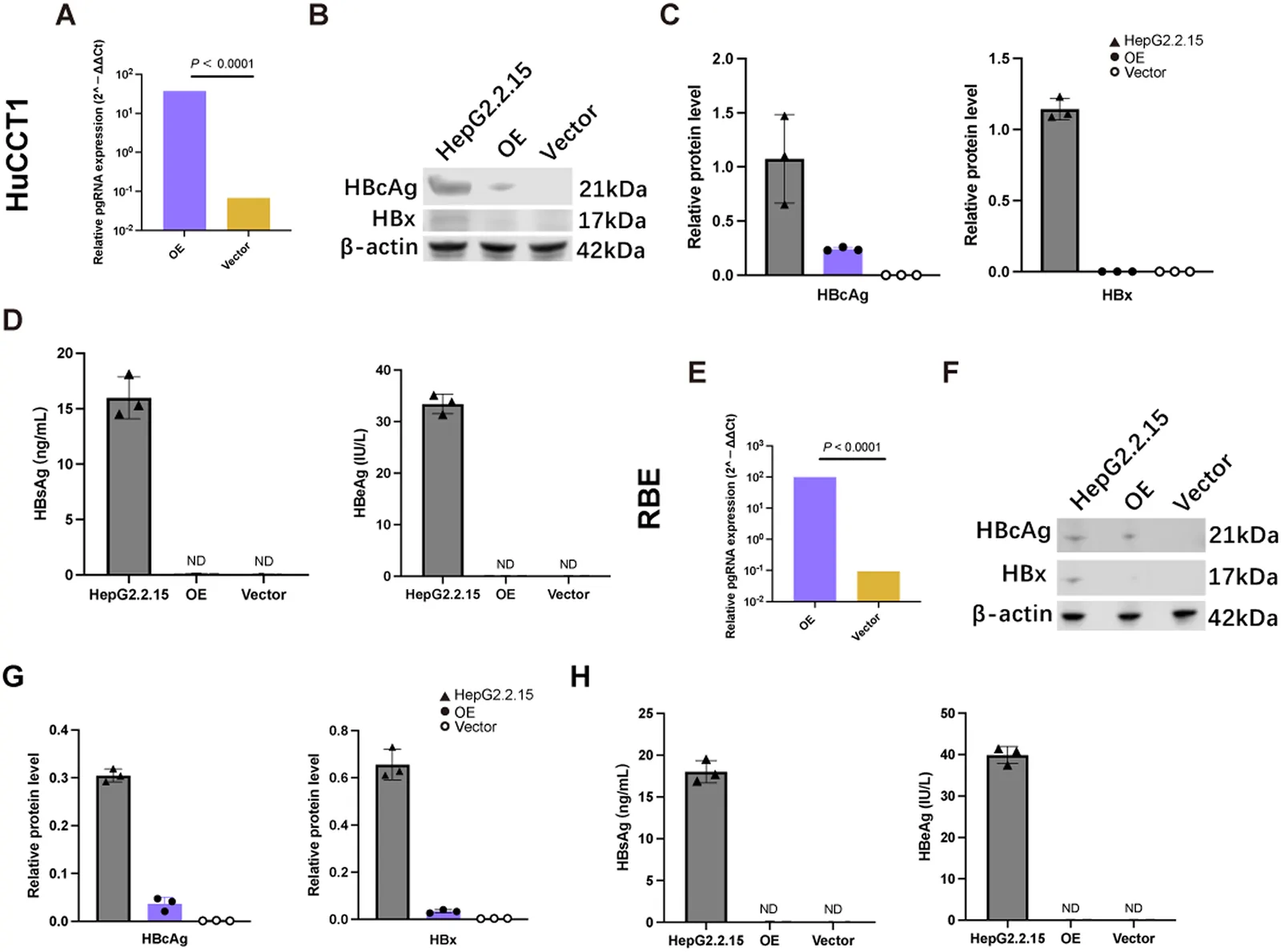
Validation of pgRNA overexpression and assessment of HBV-associated proteins and antigens in iCCA cells. (**A**, **E**) qRT-qPCR validation of pgRNA overexpression in HuCCT1 and RBE cells, respectively. (**B**, **F**) Representative western blots of HBcAg and HBx, with HepG2.2.15 cells as a positive control. (**C**, **G**) Densitometric quantification of HBcAg and HBx normalized to β-actin. (**D**, **H**) HBsAg and HBeAg levels in cell culture supernatants measured by ELISA. Data are presented as mean ± SD with individual data points shown. Exact P values are indicated in the panels. OE, pgRNA overexpression; Vector, empty-vector control; ND, below the assay detection limit

Stable HBV pgRNA-overexpression (OE) and empty-vector control (Vector) models were established in HuCCT1 and RBE cells. qRT-PCR confirmed marked pgRNA overexpression in both cell lines (both *P* < 0.0001; Fig. 2A, E). Using HepG2.2.15 cells as a positive control, western blotting detected low-level HBcAg in OE cells, whereas HBx remained minimal or undetectable (Fig. 2B, C, F, G). HBsAg and HBeAg concentrations in OE and Vector culture supernatants were below the respective ELISA detection limits (ND; Fig. 2D, H). These findings indicate that pgRNA overexpression did not reconstitute a complete HBV protein-expression or antigen-secretion profile.

#### pgRNA overexpression is associated with enhanced malignant phenotypes in iCCA cells

**Fig. 3:**
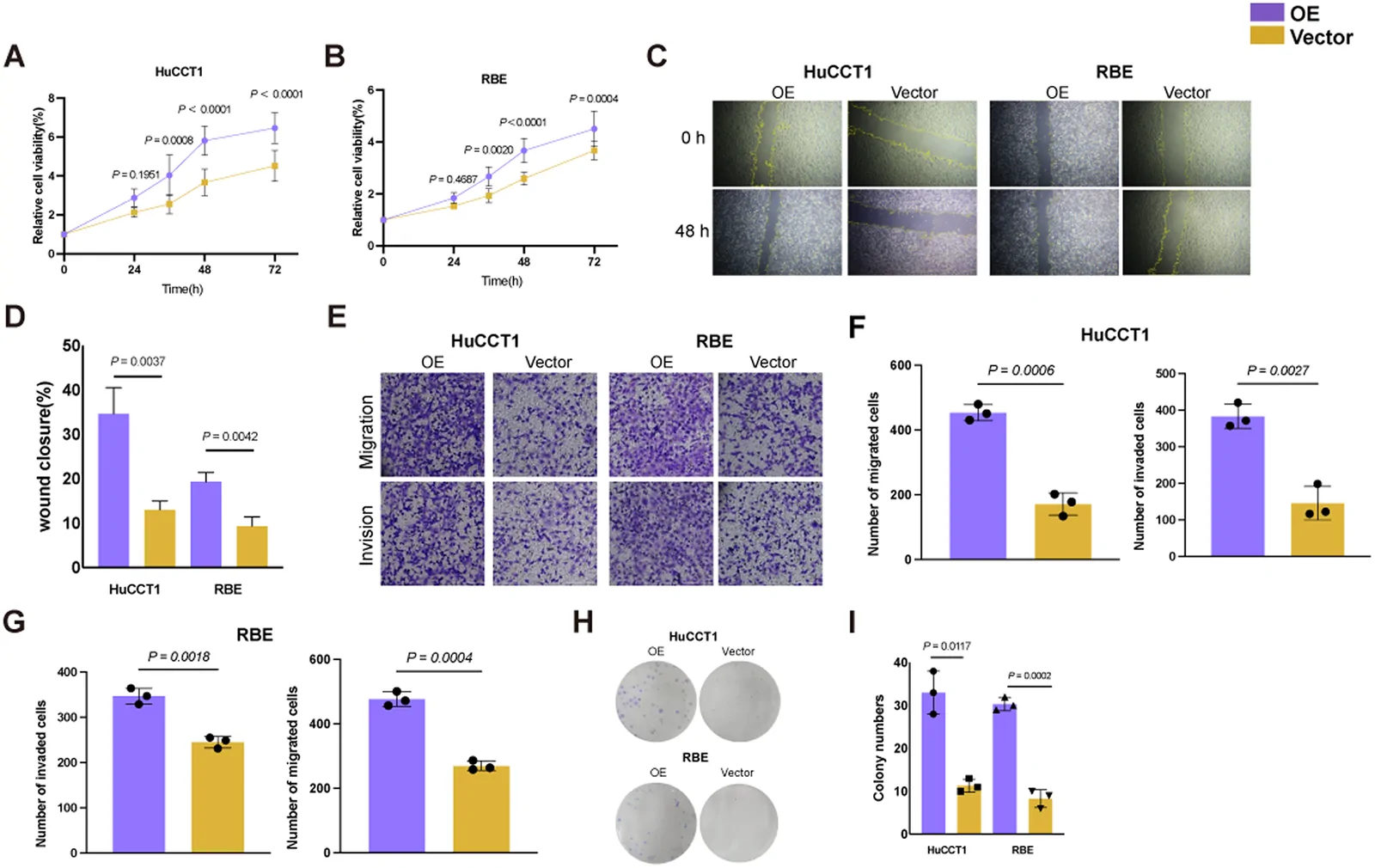
pgRNA overexpression enhances malignant phenotypes of iCCA cells. (**A**, **B**) Cell viability of pgRNA-overexpressing and vector-control HuCCT1 and RBE cells assessed by CCK-8 assay. (**C**, **D**) Representative wound-healing images and quantification of wound closure. (**E**–**G**) Representative Transwell migration and invasion images and corresponding quantification. (**H**, **I**) Representative colony-formation images and quantification. Data are presented as mean ± SD; exact *P* values are indicated in the panels. OE, pgRNA overexpression; Vector, empty-vector control

In vitro functional assays showed enhanced malignant phenotypes in both pgRNA-overexpressing cell models. CCK-8 measurements showed greater cell viability at later time points in OE cells than in Vector controls (Fig. 3A, B). Wound closure at 48 h was increased in HuCCT1 (*P* = 0.0037) and RBE (*P* = 0.0042) OE cells (Fig. 3C, D). Transwell assays showed increased migration and invasion in HuCCT1 (*P* = 0.0006 and *P* = 0.0027, respectively) and RBE cells (*P* = 0.0018 and *P* = 0.0004, respectively; Fig. 3E–G). Colony formation was also increased in HuCCT1 (*P* = 0.0117) and RBE (*P* = 0.0002) OE cells (Fig. 3H, I).

#### pgRNA overexpression is associated with upregulation of stemness-related markers

**Fig. 4:**
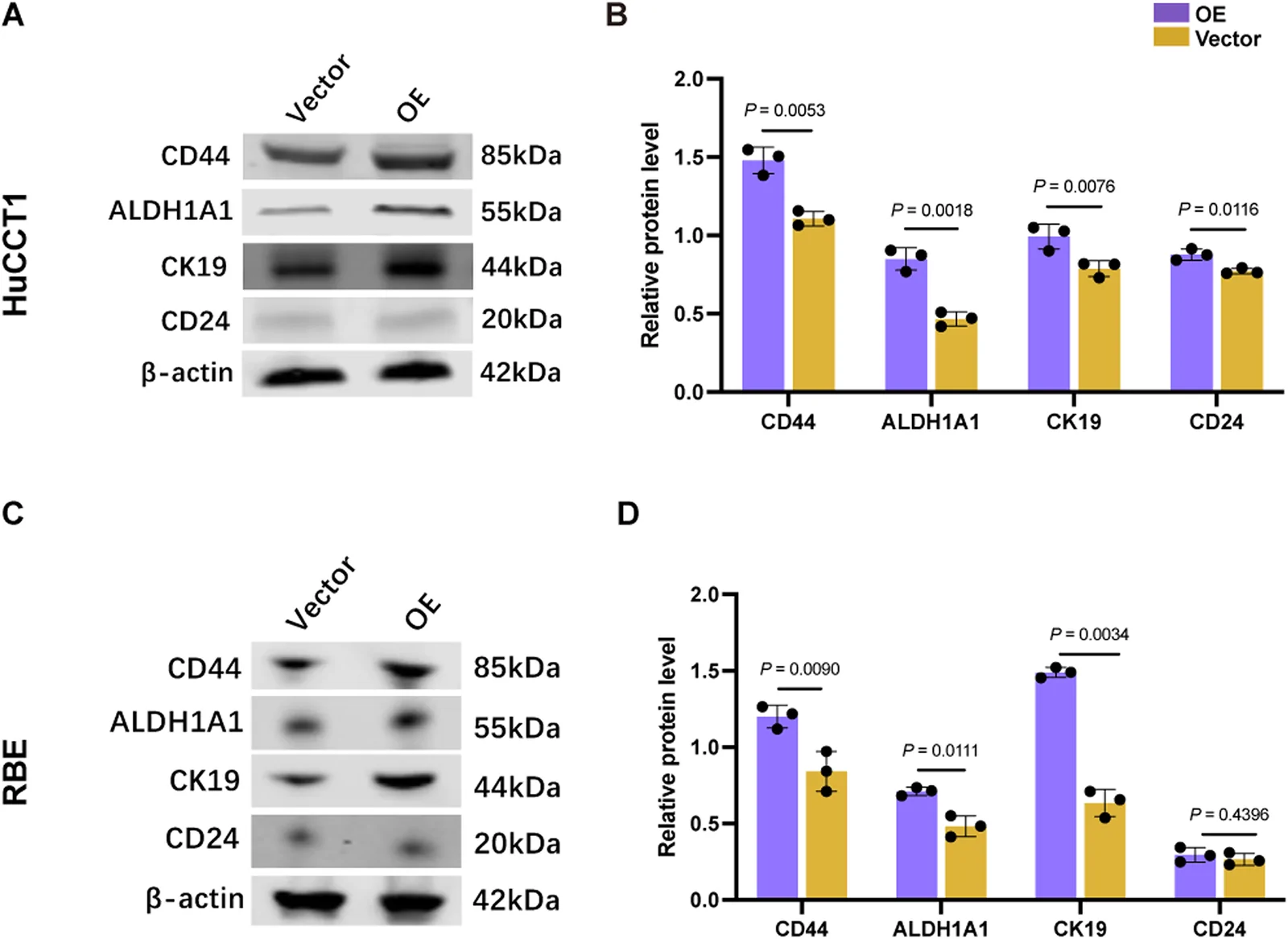
pgRNA overexpression increases multiple stemness-related proteins in iCCA cells. (**A**, **C**) Representative western blots of CD44, ALDH1A1, CK19, and CD24 in pgRNA-overexpressing and vector-control HuCCT1 and RBE cells, respectively. (**B**, **D**) Densitometric quantification of protein levels normalized to β-actin. Data are presented as mean ± SD with individual data points shown. Exact P values are indicated in the panels. OE, pgRNA overexpression; Vector, empty-vector control

Western blotting and densitometric analyses showed increased expression of multiple stemness-related proteins after pgRNA overexpression (Fig. 4). In HuCCT1 cells, CD44 (*P* = 0.0053), ALDH1A1 (*P* = 0.0018), CK19 (*P* = 0.0076) and CD24 (*P* = 0.0116) were significantly increased (Fig. 4A, B). In RBE cells, CD44 (*P* = 0.0090), ALDH1A1 (*P* = 0.0111) and CK19 (*P* = 0.0034) were significantly increased, whereas CD24 was unchanged (*P* = 0.4396; Fig. 4C, D). These findings support an association between pgRNA overexpression and a broader stemness-related protein phenotype rather than uniform induction of every marker.

### Exploratory association between preoperative serum pgRNA and postoperative outcomes

#### Preoperative serum pgRNA and overall survival

**Fig. 5:**
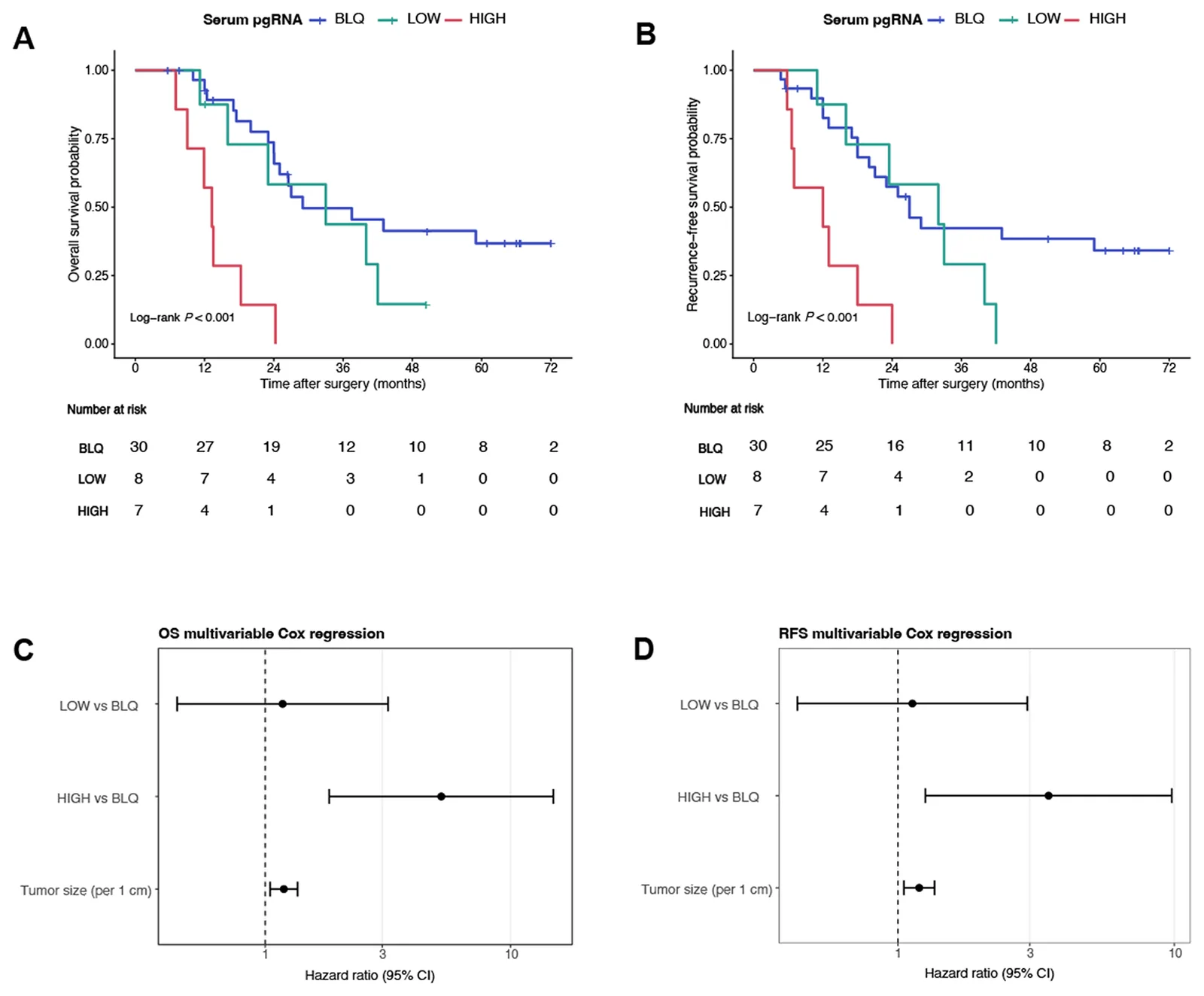
Association of serum pgRNA levels with overall and recurrence-free survival in patients with iCCA. **(A**,** B)** Kaplan–Meier curves for overall survival (OS) and recurrence-free survival (RFS), respectively, according to serum pgRNA group (BLQ, *n* = 30; LOW, *n* = 8; HIGH, *n* = 7). Differences among groups were assessed using the log-rank test, with numbers at risk shown below the curves. **(C**,** D)** Forest plots of the primary multivariable Cox proportional hazards models for OS and RFS, respectively, including serum pgRNA group and tumor size. BLQ was used as the reference category. Points indicate hazard ratios (HRs), and horizontal lines indicate 95% confidence intervals (CIs). BLQ, below the lower limit of quantification. RFS was defined as the time from surgery to the first occurrence of tumor recurrence or death from any cause, whichever occurred first

All 45 patients were included in the OS analysis, with 29 deaths during follow-up. Kaplan–Meier analysis showed a significant overall difference in OS among the three preoperative serum pgRNA groups (log-rank *P* < 0.001; Fig. 5A). The HIGH group had the poorest OS, whereas the LOW and BLQ curves were more similar. Full univariable Cox analyses of clinicopathological variables are provided in Supplementary Table S1.

Using BLQ as the reference group, univariable Cox analysis showed increased mortality risk in the HIGH group (HR = 7.59, 95% CI 2.79–20.65; *P* < 0.001), whereas the LOW group did not differ significantly from BLQ (HR = 1.65, 95% CI 0.64–4.27; *P* = 0.303). After adjustment for tumor size, the HIGH group remained associated with increased mortality risk (adjusted HR = 5.22, 95% CI 1.82–14.95; *P* = 0.002), whereas the LOW group remained non-significant (adjusted HR = 1.18, 95% CI 0.44–3.17; *P* = 0.747). Tumor size was also associated with mortality (adjusted HR per 1-cm increase = 1.19, 95% CI 1.05–1.35; *P* = 0.008; Table 2).

**Table 2:** Cox proportional hazards analyses of serum pgRNA levels for overall and recurrence-free survival

Endpoint	Variable	Univariable HR (95% CI)	*P* value	Multivariable HR (95% CI)	*P* value
OS	Serum pgRNA LOW vs. BLQ	1.65 (0.64–4.27)	0.303	1.18 (0.44–3.17)	0.747
	HIGH vs. BLQ	7.59 (2.79–20.65)	< 0.001	5.22 (1.82–14.95)	0.002
	Tumor size, per 1 cm increase	1.22 (1.09–1.37)	< 0.001	1.19 (1.05–1.35)	0.008
RFS	Serum pgRNA LOW vs. BLQ	1.64 (0.67–4.00)	0.278	1.13 (0.43–2.94)	0.806
	HIGH vs. BLQ	5.63 (2.15–14.74)	< 0.001	3.51 (1.26–9.79)	0.017
	Tumor size, per 1 cm increase	1.23 (1.10–1.37)	< 0.001	1.19 (1.05–1.36)	0.006

Abbreviations: HR, hazard ratio; CI, confidence interval; OS, overall survival; RFS, recurrence-free survival; pgRNA, pregenomic RNA; BLQ, below the lower limit of quantification

Notes: Hazard ratios (HRs) and 95% confidence intervals (CIs) were estimated using Cox proportional hazards models. BLQ was used as the reference category for serum pgRNA. The multivariable models included serum pgRNA group and tumor size. Tumor size was modeled as a continuous variable per 1-cm increase. The proportional hazards assumption was assessed using Schoenfeld residuals, with no significant global violation detected for either model. All tests were two-sided, and *P* < 0.05 was considered statistically significant. RFS was defined as the time from surgery to the first occurrence of documented tumor recurrence or death from any cause, whichever occurred first

Schoenfeld residual testing showed no significant global violation of the proportional hazards assumption for the OS model (GLOBAL *P* = 0.427; Supplementary Table S3). At 24 months, RMST was 21.92, 21.09 and 13.85 months in the BLQ, LOW and HIGH groups, respectively. Compared with BLQ, the HIGH group had an 8.07-month shorter RMST (95% CI − 12.30 to − 3.84; Holm-adjusted *P* < 0.001), whereas LOW did not differ significantly from BLQ (difference − 0.84 months; 95% CI − 4.49 to 2.81; Holm-adjusted *P* = 0.652; Supplementary Table S5).

#### Preoperative serum pgRNA and recurrence-free survival

All 45 patients were included in the RFS analysis, with 32 RFS events during follow-up. Kaplan–Meier analysis showed a significant overall difference in RFS among serum pgRNA groups (log-rank *P* < 0.001; Fig. 5B). Using BLQ as the reference, the HIGH group had a higher risk of an RFS event in univariable analysis (HR  = 5.63, 95% CI 2.15–14.74; *P* < 0.001), whereas LOW did not differ significantly from BLQ (HR = 1.64, 95% CI 0.67–4.00; *P* = 0.278). After adjustment for tumor size, HIGH remained associated with poorer RFS (adjusted HR = 3.51, 95% CI 1.26–9.79; *P* = 0.017), whereas LOW remained non-significant (adjusted HR = 1.13, 95% CI 0.43–2.94; *P* = 0.806). Tumor size was also associated with RFS (adjusted HR per 1-cm increase = 1.19, 95% CI 1.05–1.36; *P* = 0.006; Table 2).

Schoenfeld residual testing showed no significant global violation of the proportional hazards assumption for the RFS model (GLOBAL *P* = 0.119; Supplementary Table S3). At 24 months, RFS RMST was 20.01, 21.13 and 12.35 months in the BLQ, LOW and HIGH groups, respectively. Compared with BLQ, the HIGH group had a 7.67-month shorter RMST (95% CI − 12.77 to − 2.56; Holm-adjusted *P* = 0.008), whereas LOW did not differ significantly from BLQ (difference 1.12 months; 95% CI − 2.87 to 5.12; Holm-adjusted *P* = 0.582; Supplementary Table S5).

Sensitivity analyses supported the primary serum pgRNA findings (Supplementary Table S4). When HIGH was compared with the combined BLQ/LOW group after adjustment for tumor size, the HR was 4.95 for OS (95% CI 1.82–13.48; *P* = 0.002) and 3.35 for RFS (95% CI 1.29–8.75; *P* = 0.013). Modeled continuously, each two-fold increase in serum pgRNA was associated with a higher risk of death (adjusted HR = 1.16, 95% CI 1.07–1.26; *P* < 0.001) and a higher risk of an RFS event (adjusted HR = 1.14, 95% CI 1.05–1.25; *P* = 0.003). In contrast, detectable (LOW/HIGH) versus BLQ status was not significant for either endpoint. Additional adjustment for CA19-9 and/or AJCC stage did not eliminate the adverse association of the HIGH group.

Exploratory tissue pgRNA outcome analyses are shown in Supplementary Fig. S3 and Supplementary Tables S6–S7. Conventional tumor-tissue pgRNA Cox models violated the proportional hazards assumption (OS *P* = 0.011; RFS *P* = 0.005). Tumor-size-adjusted time-varying models suggested that the adverse association per two-fold increase in tumor-tissue pgRNA was strongest early after surgery and attenuated over time (*P* for time interaction = 0.018 for OS and 0.010 for RFS); at 12 months, the estimated HR was 1.27 for both OS (95% CI 1.01–1.61; *P* = 0.045) and RFS (95% CI 1.05–1.54; *P* = 0.016). Adjacent non-tumor tissue pgRNA was associated with OS and RFS in univariable models but was not significant after adjustment for tumor size (Supplementary Table S6).

## Discussion

The biological and clinical relevance of residual HBV transcription-associated signals in HBV-related iCCA remains poorly defined. In this exploratory translational study, we examined HBV pgRNA at the levels of tissue localization, cell phenotype and postoperative outcomes. Overall pgRNA expression was lower in tumor tissues than in paired adjacent non-tumor liver tissues, while pgRNA signals remained detectable in focal CK19-positive tumor epithelial regions. In two iCCA cell lines, stable pgRNA overexpression was associated with enhanced malignant phenotypes and increased expression of multiple stemness-related proteins. Clinically, the serum pgRNA HIGH group was associated with poorer OS and RFS after adjustment for tumor size. Together, these findings support a hypothesis-generating association between persistent HBV transcription-associated signals and aggressive tumor behavior in HBV-related iCCA rather than establishing a direct causal role of pgRNA itself.

The tissue findings showed that, although overall pgRNA expression was lower in tumor tissues than in adjacent non-tumor liver tissues, pgRNA signals were still detectable in focal CK19-positive tumor epithelial regions. This pattern may reflect spatial heterogeneity of HBV transcriptional activity within tumor tissues. HBV DNA in iCCA tumor cells may be present predominantly in integrated forms, with impaired completion of the full viral replication cycle, resulting in lower overall pgRNA levels than in adjacent non-tumor liver tissue. Conversely, some tumor cells or their neighboring microenvironment may retain residual viral transcriptional activity. Previous genomic analyses have documented HBV integration and associated genomic aberrations in HBV-related iCCA [8]. Integrated HBV DNA can also produce viral RNAs in hepatocellular carcinoma, indicating that tumor-associated HBV RNA signals cannot be attributed solely to cccDNA transcription [11]. Importantly, the IF–FISH data provide descriptive spatial evidence only: they do not demonstrate that pgRNA originates from cccDNA transcription within tumor cells and cannot distinguish cccDNA-derived transcripts from transcripts associated with integrated HBV sequences. These questions require orthogonal validation using RNase-treated and scramble-probe controls, laser-capture microdissection, single-cell spatial transcriptomics, cccDNA quantification or HBV integration-site analysis.

Paired tissue analysis showed lower pgRNA levels in tumor tissues than in adjacent non-tumor liver tissues, while serum pgRNA remained detectable in a subset of patients. Serum-defined BLQ, LOW and HIGH groups did not show significant differences in tumor or adjacent-tissue pgRNA expression. Among the 15 patients with quantifiable serum pgRNA, serum pgRNA was not significantly correlated with tumor-tissue pgRNA but showed a moderate positive correlation with adjacent non-tumor tissue pgRNA. Given the small sample, this finding is exploratory but is compatible with the concept that circulating HBV RNA may partly reflect residual transcriptional activity in non-tumor liver during NA therapy [9, 10]. Thus, serum pgRNA should not be interpreted as a direct surrogate for tumor-tissue pgRNA expression.

In vitro experiments in HuCCT1 and RBE cells showed that pgRNA overexpression was associated with enhanced malignant phenotypes and increased expression of multiple stemness-related proteins. In HBV-related HCC, pgRNA has been linked to tumor stemness and progression through mechanisms involving IGF2BP3 [14]. iCCA stemness phenotypes have also been associated with CD44, ALDH1A1, CK19 and CD24 [15, 16], as well as Wnt/β-catenin and Hippo/YAP signaling [17]. The concordant increases in CD44, ALDH1A1 and CK19 across both cell lines support a stemness-related phenotype, whereas the lack of a significant CD24 change in RBE cells illustrates marker-specific heterogeneity. Nevertheless, the study used gain-of-function models only, without pgRNA knockdown, rescue experiments or in vivo validation; therefore, causality and the responsible molecular pathway remain unresolved.

From a translational perspective, pgRNA has viral specificity and may complement established prognostic factors such as CA19-9, tumor size and AJCC stage by providing information on residual HBV transcriptional activity [9, 10]. CA19-9 and carcinoembryonic antigen are commonly evaluated serum biomarkers in iCCA, but their prognostic performance is imperfect [28]. In this cohort, CA19-9 did not differ significantly across serum pgRNA groups, yet CA19-9 itself was prognostic in univariable analyses, and adjustment for CA19-9 did not remove the adverse association of the HIGH pgRNA group. The primary tumor-size-adjusted models likewise showed poorer OS and RFS in the HIGH group. These findings support serum pgRNA as a candidate complementary prognostic biomarker, but external validation is required before clinical implementation.

The association with RFS was directionally consistent with the OS findings. The HIGH group had poorer RFS in the primary Cox model, and the 24-month RMST analysis also showed substantially shorter RFS in HIGH than BLQ. However, the HIGH group contained only seven patients and its risk set declined rapidly during follow-up, so late survival estimates and effect sizes remain imprecise. RFS estimates in retrospective cohorts may also be influenced by surveillance frequency, imaging intervals, postoperative treatments and heterogeneity in the causes and timing of endpoint events. The RFS findings should therefore be interpreted as supportive evidence requiring external validation rather than as a clinically established prognostic effect.

Previous studies in HBV-related HCC have reported associations between pgRNA levels in tumor tissue, adjacent non-tumor liver tissue or serum and tumor stemness [13, 14], recurrence risk [13] and prognosis [12]. The present study extends this hypothesis to HBV-related iCCA but should not be interpreted as evidence that the biological significance of pgRNA is shared across tumor types. iCCA and HCC differ in cell of origin, molecular subtype, tumor microenvironment, HBV integration pattern and clinical course [1, 29]. iCCA also exhibits molecular and etiological heterogeneity [2, 4]. Future studies should jointly evaluate tumor-tissue pgRNA, adjacent non-tumor liver pgRNA, serum pgRNA, HBV DNA integration status and cccDNA activity in adequately powered HBV-related iCCA cohorts.

Clinically, the present findings suggest that preoperative serum pgRNA may help identify a subgroup of HBV-related iCCA patients with adverse postoperative outcomes when considered together with tumor burden and other clinicopathological variables. However, the limited sample size and single-center retrospective design preclude clinical decision-making based on pgRNA alone. Larger prospective multicenter cohorts should test whether pgRNA improves risk stratification beyond CA19-9, AJCC stage, tumor size and other established biomarkers [28].

This study has several limitations. First, it was a single-center retrospective study with only 45 patients, including seven in the HIGH group, which limits precision and generalizability. Second, the serum pgRNA cutoff was data-driven, and the small subgroup sizes and rapidly diminishing late risk sets require external validation of both OS and RFS estimates. Third, the IF–FISH analysis included only five archival cases and lacked RNase-treated and scramble-probe controls, so it provides descriptive spatial evidence without resolving the intracellular source of pgRNA. Fourth, the in vitro experiments used two gain-of-function iCCA cell models but lacked pgRNA knockdown, rescue experiments and in vivo validation. Fifth, HBV integration sites, cccDNA, HBcrAg and detailed antiviral-treatment dynamics were not systematically integrated. Sixth, serum–tissue correlation analyses were limited to 15 patients with quantifiable serum pgRNA, and tumor-tissue pgRNA showed time-varying rather than proportional survival associations. These limitations preclude causal inference and require confirmation in larger prospective multicenter studies.

In conclusion, HBV pgRNA was detectable in focal CK19-positive tumor epithelial regions and was associated with malignant and stemness-related phenotypes in two iCCA cell models. In patients receiving long-term NA therapy with suppressed serum HBV DNA, high preoperative serum pgRNA was associated with poorer postoperative OS and RFS. These exploratory findings support further mechanistic investigation and prospective multicenter validation of pgRNA as a candidate infection-related biomarker in HBV-related iCCA.

## Supplementary Information

Below is the link to the electronic supplementary material.

Supplementary Material 1

Supplementary Material 2

## Data Availability

All experimental data supporting the findings of this study are available within the article and its Supplementary Information. The clinical datasets generated and/or analysed during the current study are not publicly available because they contain potentially identifiable patient information and are subject to institutional and ethical restrictions. De-identified clinical data are available from the corresponding authors upon reasonable request, subject to approval by the relevant ethics committee and institution.

## Abbreviations

AFP: Alpha-fetoprotein
AJCC: American Joint Committee on Cancer
BLQ: Below the lower limit of quantification
CA19-9: Carbohydrate antigen 19 − 9
cccDNA: Covalently closed circular DNA
CI: Confidence interval
CK19: Cytokeratin 19
HBcAg: Hepatitis B core antigen
HBeAg: Hepatitis B e antigen
HBsAg: Hepatitis B surface antigen
HBV: Hepatitis B virus
HBx: Hepatitis B virus X protein
HR: Hazard ratio
iCCA: Intrahepatic cholangiocarcinoma
IF-FISH: Immunofluorescence–fluorescence in situ hybridization
NA: Nucleos(t)ide analogue
OS: Overall survival
pgRNA: Pregenomic RNA
qRT-PCR: Quantitative reverse-transcription polymerase chain reaction
RFS: Recurrence-free survival
RMST: Restricted mean survival time
SAT: Simultaneous amplification and testing
SD: Standard deviation
