# Supplementary material for: Hepatitis B virus pregenomic RNA is associated with malignant phenotypes and postoperative survival in HBV-related intrahepatic cholangiocarcinoma
Source: Infect Agent Cancer. 2026 Sep 10;21:60. doi: 10.1186/s13027-026-00792-1 (PMC13573472; doi:10.1186/s13027-026-00792-1)

Fig.2B Uncropped western blots

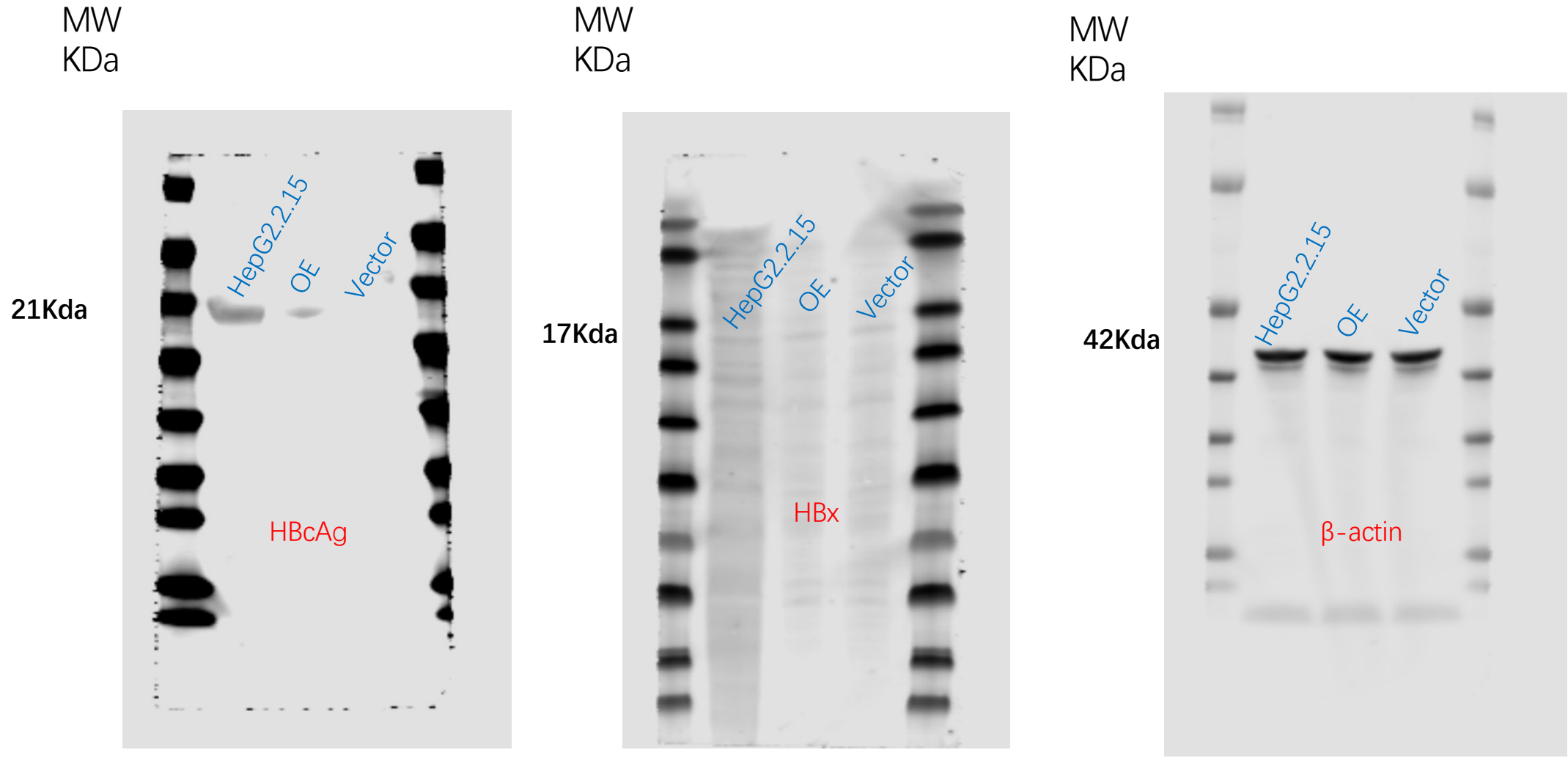

Fig.2F Uncropped western blots

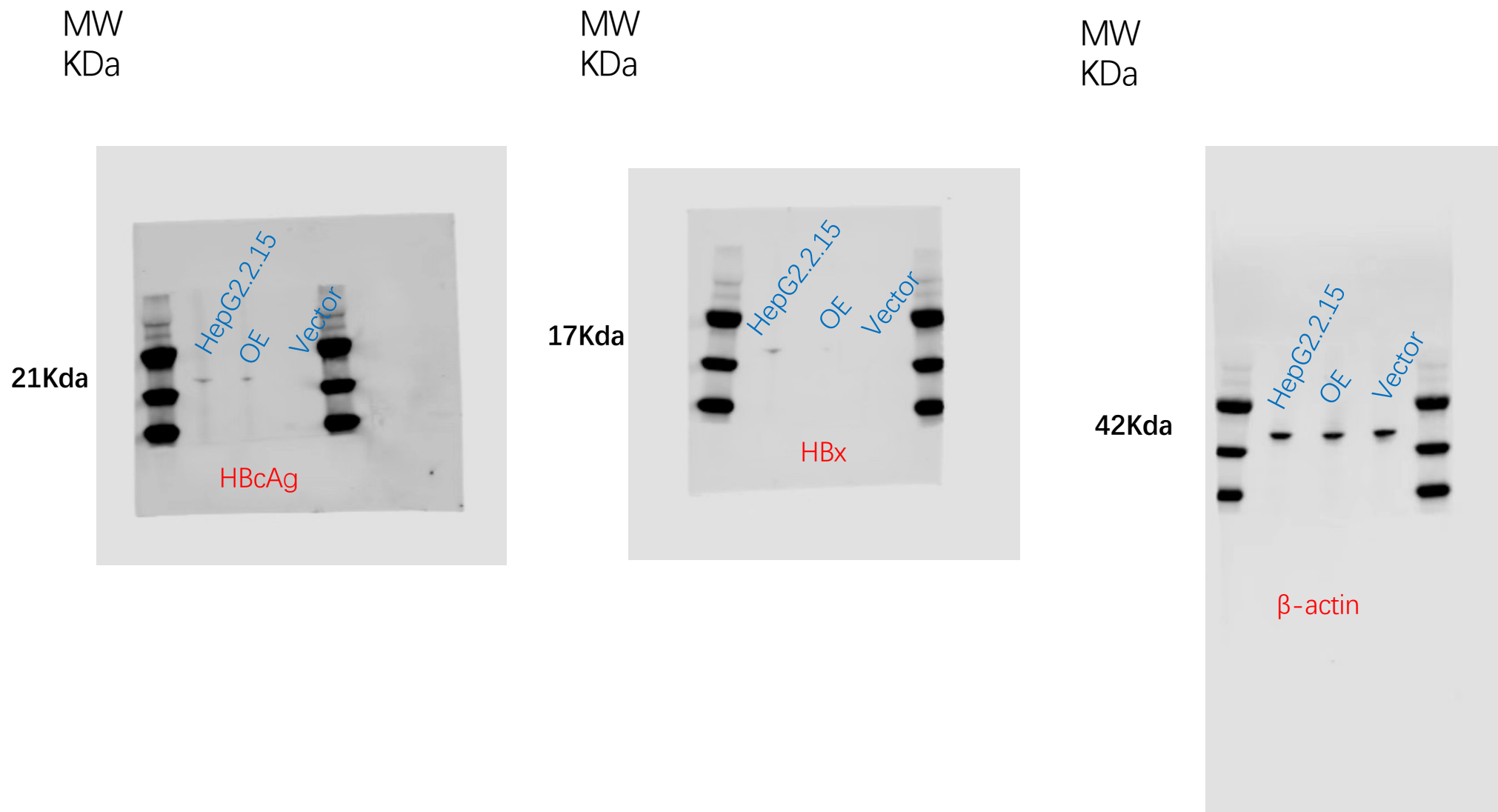

Fig.4A Uncropped western blots

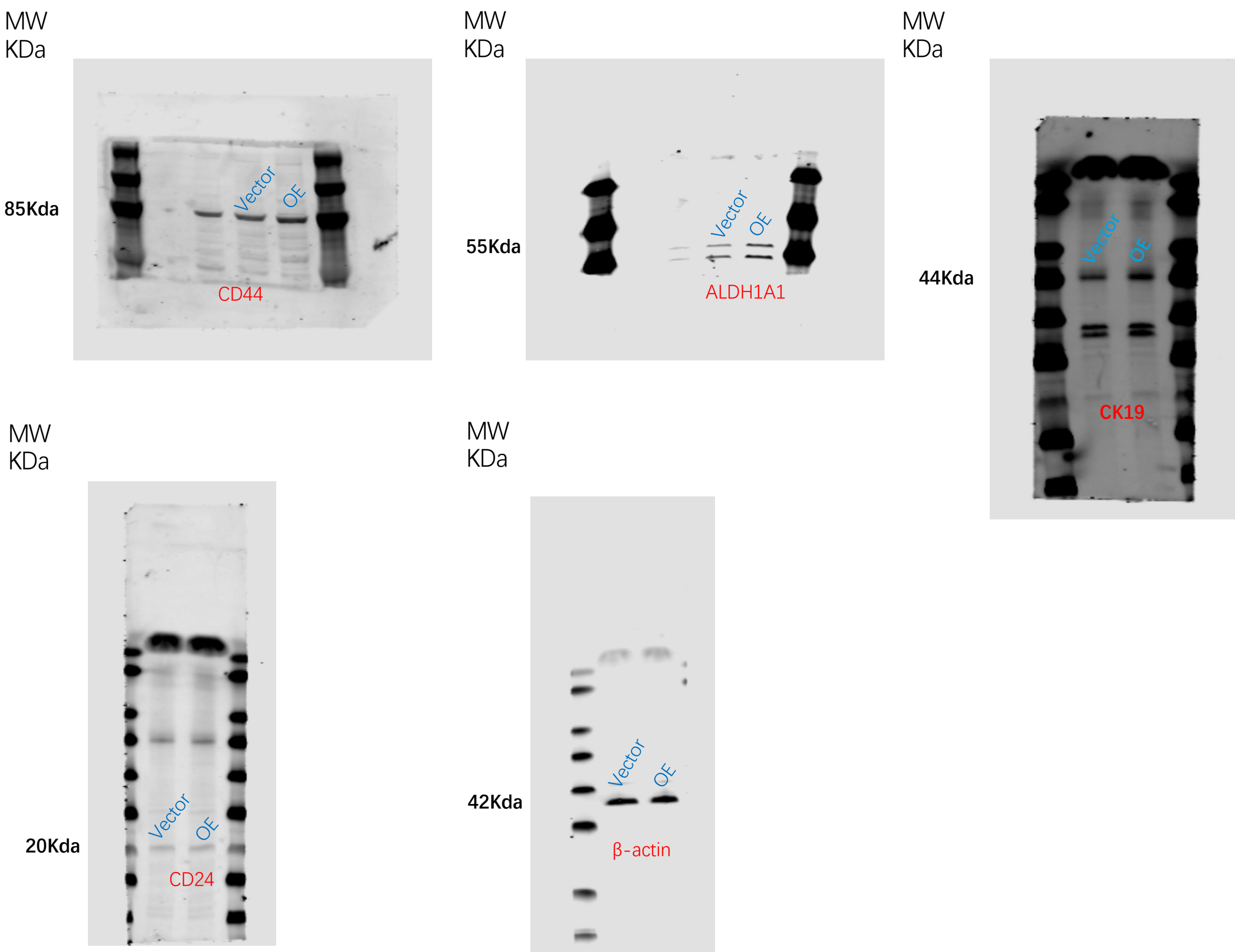

Fig.4C Uncropped western blots

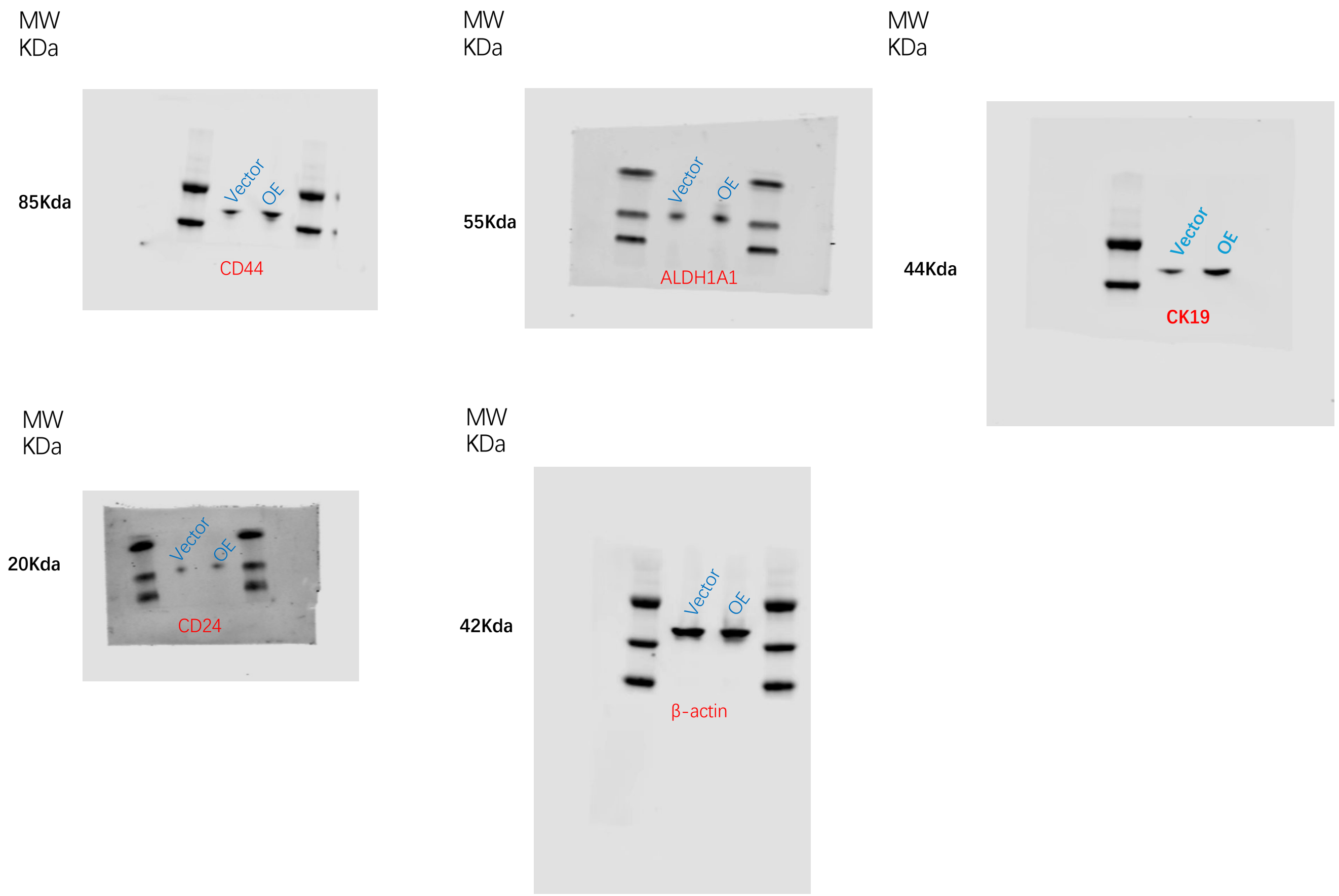

Supplement: Supplementary file 1 [file 13027_2026_792_MOESM1_ESM.pdf]
