# Supplementary material for: Hepatitis B virus pregenomic RNA is associated with malignant phenotypes and postoperative survival in HBV-related intrahepatic cholangiocarcinoma
Source: Infect Agent Cancer. 2026 Sep 10;21:60. doi: 10.1186/s13027-026-00792-1 (PMC13573472; doi:10.1186/s13027-026-00792-1)
Supplement: Supplementary file 2 [file 13027_2026_792_MOESM2_ESM.docx]

Supplementary Figure S1. Flow diagram of patient screening and cohort selection.

A total of 287 patients were screened, and 45 patients were included in the final analytic cohort according to the predefined eligibility criteria. HBsAg, hepatitis B surface antigen; HBV, hepatitis B virus; HCV, hepatitis C virus; HDV, hepatitis D virus; HIV, human immunodeficiency virus; NA, nucleos(t)ide analogue.

Supplementary Figure S2. Associations between serum pgRNA and tissue levels in HBV-related iCCA.

(A,B) Tumor (A) and adjacent non-tumor tissue (B) pgRNA expression across serum pgRNA groups. Relative expression was calculated using the 2^−ΔCt method and displayed on the log₁₀ scale; group comparisons were performed on ΔCt values using Welch’s one-way ANOVA. (C,D) Spearman correlations of serum pgRNA with tumor (C) and adjacent non-tumor tissue (D) pgRNA expression among patients with quantifiable serum pgRNA (n = 15). Each point represents one patient. BLQ, below the lower limit of quantification.

Supplementary Figure S3. Time-varying associations between tumor-tissue pgRNA expression and postoperative outcomes.

(A,C) Scaled Schoenfeld residual plots for tumor-tissue pgRNA in tumor-size-adjusted Cox models for OS (A) and RFS (C); the proportional hazards assumption was violated for tumor-tissue pgRNA (OS, *P* = 0.011; RFS, *P* = 0.005). (B,D) Time-varying HRs for OS (B) and RFS (D) per two-fold increase in tumor-tissue pgRNA expression, adjusted for tumor size. Time interactions were significant for OS (*P* = 0.018) and RFS (*P* = 0.010). Shaded areas indicate 95% CIs and dashed lines indicate HR = 1.RFS was defined as the interval from surgery to the first occurrence of documented recurrence or death from any cause, whichever occurred first; patients alive and recurrence-free were censored at their last valid recurrence-free follow-up.

Supplementary Table S1. Univariable Cox proportional hazards analyses for overall and recurrence-free survival.

| Variable | OS HR (95% CI) | *P* value | RFS HR (95% CI) | *P* value |
| --- | --- | --- | --- | --- |
| Serum pgRNA group |  |  |  |  |
| LOW vs BLQ | 1.65 (0.64–4.27) | 0.303 | 1.64 (0.67–4.00) | 0.278 |
| HIGH vs BLQ | 7.59 (2.79–20.65) | <0.001 | 5.63 (2.15–14.74) | <0.001 |
| Age, per year | 1.02 (0.97–1.07) | 0.405 | 1.00 (0.96–1.05) | 0.872 |
| Male vs female | 1.33 (0.54–3.27) | 0.535 | 1.51 (0.62–3.66) | 0.368 |
| Tumor size, per 1-cm increase | 1.22 (1.09–1.37) | <0.001 | 1.23 (1.10–1.37) | <0.001 |
| Multiple vs solitary tumors | 1.59 (0.75–3.41) | 0.229 | 1.45 (0.69–3.03) | 0.324 |
| T category |  |  |  |  |
| T2 vs T1 | 1.59 (0.60–4.22) | 0.356 | 1.36 (0.53–3.48) | 0.525 |
| T3 vs T1 | 4.80 (1.78–12.92) | 0.002 | 4.20 (1.68–10.53) | 0.002 |
| T4 vs T1 | 7.05 (2.19–22.71) | 0.001 | 7.64 (2.44–23.96) | <0.001 |
| N1 vs N0 | 2.37 (0.92–6.10) | 0.073 | 2.29 (0.91–5.77) | 0.079 |
| AJCC 8th edition stage |  |  |  |  |
| Stage II vs Stage I | 1.56 (0.50–4.83) | 0.440 | 1.28 (0.43–3.76) | 0.659 |
| Stage III vs Stage I | 5.78 (2.33–14.32) | <0.001 | 4.83 (2.10–11.07) | <0.001 |
| AFP, per doubling | 0.91 (0.71–1.16) | 0.439 | 0.86 (0.67–1.10) | 0.234 |
| CA19-9, per doubling | 1.32 (1.11–1.57) | 0.001 | 1.25 (1.06–1.47) | 0.007 |

**Abbreviations:** OS, overall survival; RFS, recurrence-free survival; HR, hazard ratio; CI, confidence interval; pgRNA, pregenomic RNA; BLQ, below the lower limit of quantification; AJCC, American Joint Committee on Cancer; AFP, alpha-fetoprotein; CA19-9, carbohydrate antigen 19-9.

**Notes:** Reference categories were BLQ, female sex, solitary tumor, T1, N0, and AJCC stage I, as applicable. Tumor size was modeled per 1-cm increase; AFP and CA19-9 were modeled on the log₂ scale and are expressed per doubling.

Supplementary Table S2. Serum pgRNA characteristics of the five patients included in the IF-FISH analysis.

| IF-FISH case | Serum pgRNA (copies/mL) | Serum pgRNA stratum |
| --- | --- | --- |
| Case 1 | <100 | BLQ |
| Case 2 | 20040 | HIGH |
| Case 3 | 1924000 | HIGH |
| Case 4 | 552 | LOW |
| Case 5 | 206 | LOW |

**Note:** Patient identifiers were anonymized using study case numbers. BLQ, <100 copies/mL; LOW, 100–2700 copies/mL; HIGH, >2700 copies/mL. The representative IF-FISH image in Fig. 1A was obtained from Case 3 (HIGH).

Supplementary Table S3. Tests of the proportional hazards assumption for the primary multivariable Cox models.

| Variable | df | OS *χ²* | OS *P* value | RFS *χ²* | RFS *P* value |
| --- | --- | --- | --- | --- | --- |
| Serum pgRNA group | 2 | 0.475 | 0.788 | 5.561 | 0.062 |
| Tumor size | 1 | 1.346 | 0.246 | 0.138 | 0.710 |
| Global test | 3 | 2.780 | 0.427 | 5.855 | 0.119 |

**Abbreviations:** OS, overall survival; RFS, recurrence-free survival; pgRNA, pregenomic RNA.

**Notes:** The proportional hazards assumption was assessed using Schoenfeld residuals. The serum pgRNA group term represents a joint 2-df test of LOW and HIGH versus BLQ.

Supplementary Table S4. Sensitivity analyses of the association between serum pgRNA and overall and recurrence-free survival.

| Endpoint | Sensitivity model | Variable | HR (95% CI) | *P* value |
| --- | --- | --- | --- | --- |
| OS | HIGH vs BLQ/LOW + tumor size | HIGH vs BLQ/LOW | 4.95 (1.82–13.48) | 0.002 |
|  |  | Tumor size, per 1-cm increase | 1.20 (1.06–1.36) | 0.004 |
|  | Detectable vs BLQ + tumor size | Detectable (LOW/HIGH) vs BLQ | 1.94 (0.87–4.32) | 0.104 |
|  |  | Tumor size, per 1-cm increase | 1.18 (1.05–1.33) | 0.006 |
|  | Continuous serum pgRNA + tumor size | Serum pgRNA, per doubling | 1.16 (1.07–1.26) | <0.001 |
|  |  | Tumor size, per 1-cm increase | 1.17 (1.04–1.33) | 0.011 |
|  | Serum pgRNA group + AJCC stage | LOW vs BLQ | 2.88 (1.02–8.10) | 0.045 |
|  |  | HIGH vs BLQ | 7.18 (2.51–20.48) | <0.001 |
|  |  | AJCC stage III vs I–II | 5.95 (2.46–14.42) | <0.001 |
|  | Serum pgRNA group + CA19-9 | LOW vs BLQ | 1.40 (0.53–3.75) | 0.499 |
|  |  | HIGH vs BLQ | 7.35 (2.63–20.51) | <0.001 |
|  |  | CA19-9, per doubling | 1.32 (1.10–1.58) | 0.002 |
|  | Serum pgRNA group + tumor size + CA19-9 | LOW vs BLQ | 1.26 (0.48–3.32) | 0.637 |
|  |  | HIGH vs BLQ | 5.04 (1.71–14.85) | 0.003 |
|  |  | Tumor size, per 1-cm increase | 1.19 (1.05–1.36) | 0.007 |
|  |  | CA19-9, per doubling | 1.33 (1.11–1.60) | 0.002 |
|  | Continuous serum pgRNA + tumor size + CA19-9 | Serum pgRNA, per doubling | 1.13 (1.04–1.23) | 0.004 |
|  |  | Tumor size, per 1-cm increase | 1.19 (1.05–1.35) | 0.008 |
|  |  | CA19-9, per doubling | 1.28 (1.07–1.54) | 0.008 |
| RFS | HIGH vs BLQ/LOW + tumor size | HIGH vs BLQ/LOW | 3.35 (1.29–8.75) | 0.013 |
|  |  | Tumor size, per 1-cm increase | 1.20 (1.06–1.35) | 0.003 |
|  | Detectable vs BLQ + tumor size | Detectable (LOW/HIGH) vs BLQ | 1.67 (0.75–3.69) | 0.208 |
|  |  | Tumor size, per 1-cm increase | 1.19 (1.06–1.34) | 0.004 |
|  | Continuous serum pgRNA + tumor size | Serum pgRNA, per doubling | 1.14 (1.05–1.25) | 0.003 |
|  |  | Tumor size, per 1-cm increase | 1.17 (1.04–1.32) | 0.010 |
|  | Serum pgRNA group + AJCC stage | LOW vs BLQ | 2.61 (1.01–6.74) | 0.048 |
|  |  | HIGH vs BLQ | 5.41 (2.02–14.46) | <0.001 |
|  |  | AJCC stage III vs I–II | 5.22 (2.33–11.68) | <0.001 |
|  | Serum pgRNA group + CA19-9 | LOW vs BLQ | 1.43 (0.57–3.55) | 0.447 |
|  |  | HIGH vs BLQ | 5.90 (2.24–15.56) | <0.001 |
|  |  | CA19-9, per doubling | 1.27 (1.06–1.50) | 0.008 |
|  | Serum pgRNA group + tumor size + CA19-9 | LOW vs BLQ | 1.15 (0.45–2.91) | 0.769 |
|  |  | HIGH vs BLQ | 3.78 (1.35–10.54) | 0.011 |
|  |  | Tumor size, per 1-cm increase | 1.20 (1.05–1.36) | 0.006 |
|  |  | CA19-9, per doubling | 1.28 (1.07–1.54) | 0.008 |
|  | Continuous serum pgRNA + tumor size + CA19-9 | Serum pgRNA, per doubling | 1.14 (1.04–1.24) | 0.004 |
|  |  | Tumor size, per 1-cm increase | 1.18 (1.04–1.33) | 0.009 |
|  |  | CA19-9, per doubling | 1.25 (1.05–1.48) | 0.012 |

**Abbreviations:** OS, overall survival; RFS, recurrence-free survival; HR, hazard ratio; CI, confidence interval; pgRNA, pregenomic RNA; BLQ, below the lower limit of quantification; AJCC, American Joint Committee on Cancer; CA19-9, carbohydrate antigen 19-9.

**Notes:** BLQ was the reference category for categorical pgRNA models. HIGH vs BLQ/LOW compares HIGH with the combined BLQ and LOW groups; detectable vs BLQ compares LOW/HIGH with BLQ. Serum pgRNA and CA19-9 were modeled on the log₂ scale; tumor size was modeled per 1-cm increase.

Supplementary Table S5. Restricted mean survival time analyses at 18 and 24 months.

| Endpoint | *τ* (months) | Comparison | RMST, first group (95% CI) | RMST, second group (95% CI) | Difference, second − first (95% CI) | Raw *P* value | Holm-adjusted *P* value |
| --- | --- | --- | --- | --- | --- | --- | --- |
| OS | 18 | BLQ vs LOW | 17.23 (16.47–18.00) | 16.86 (15.28–18.43) | -0.38 (-2.13–1.37) | 0.672 | 0.672 |
| OS | 18 | BLQ vs HIGH | 17.23 (16.47–18.00) | 12.95 (10.11–15.80) | -4.28 (-7.23– -1.33) | 0.004 | 0.013 |
| OS | 18 | LOW vs HIGH | 16.86 (15.28–18.43) | 12.95 (10.11–15.80) | -3.90 (-7.16– -0.65) | 0.019 | 0.038 |
| RFS | 18 | BLQ vs LOW | 16.21 (14.86–17.55) | 16.83 (15.22–18.45) | 0.63 (-1.47–2.73) | 0.559 | 0.559 |
| RFS | 18 | BLQ vs HIGH | 16.21 (14.86–17.55) | 11.49 (7.91–15.07) | -4.72 (-8.54– -0.89) | 0.016 | 0.031 |
| RFS | 18 | LOW vs HIGH | 16.83 (15.22–18.45) | 11.49 (7.91–15.07) | -5.34 (-9.27– -1.41) | 0.008 | 0.023 |
| OS | 24 | BLQ vs LOW | 21.92 (20.38–23.47) | 21.09 (17.78–24.39) | -0.84 (-4.49–2.81) | 0.652 | 0.652 |
| OS | 24 | BLQ vs HIGH | 21.92 (20.38–23.47) | 13.85 (9.92–17.79) | -8.07 (-12.30– -3.84) | <0.001 | <0.001 |
| OS | 24 | LOW vs HIGH | 21.09 (17.78–24.39) | 13.85 (9.92–17.79) | -7.23 (-12.37– -2.09) | 0.006 | 0.012 |
| RFS | 24 | BLQ vs LOW | 20.01 (17.85–22.17) | 21.13 (17.77–24.50) | 1.12 (-2.87–5.12) | 0.582 | 0.582 |
| RFS | 24 | BLQ vs HIGH | 20.01 (17.85–22.17) | 12.35 (7.72–16.97) | -7.67 (-12.77– -2.56) | 0.003 | 0.008 |
| RFS | 24 | LOW vs HIGH | 21.13 (17.77–24.50) | 12.35 (7.72–16.97) | -8.79 (-14.50– -3.07) | 0.003 | 0.008 |

**Abbreviations:** OS, overall survival; RFS, recurrence-free survival; RMST, restricted mean survival time; CI, confidence interval; BLQ, below the lower limit of quantification.

**Notes:** RMST was estimated at truncation times of 18 and 24 months. RMST differences were calculated as the second-listed group minus the first-listed group. *P* values were adjusted for the three pairwise comparisons within each endpoint and truncation time using the Holm method.

Supplementary Table S6. Conventional Cox regression analyses of tissue pgRNA expression and postoperative outcomes.

| Tissue source | Endpoint | Univariable HR (95% CI) | *P* | Adjusted HR (95% CI)† | *P* | PH test for pgRNA, *P* | Global PH test, *P* |
| --- | --- | --- | --- | --- | --- | --- | --- |
| Tumor tissue | OS | 1.05 (0.91–1.20) | 0.523 | 1.02 (0.89–1.17) | 0.779 | 0.011 | 0.021 |
| Tumor tissue | RFS | 1.12 (0.98–1.28) | 0.108 | 1.08 (0.94–1.23) | 0.271 | 0.005 | 0.019 |
| Adjacent non-tumor tissue | OS | 1.30 (1.03–1.66) | 0.030 | 1.25 (0.95–1.66) | 0.117 | 0.826 | 0.263 |
| Adjacent non-tumor tissue | RFS | 1.29 (1.02–1.62) | 0.030 | 1.24 (0.95–1.61) | 0.112 | 0.479 | 0.767 |

**Notes:** HRs are expressed per two-fold increase in tissue pgRNA expression. Adjusted models included tumor size. The PH assumption was assessed using Schoenfeld residuals. Because the PH assumption was violated for tumor-tissue pgRNA, conventional Cox estimates should not be interpreted as constant effects over follow-up; time-varying analyses are shown in Supplementary Table S7.

Supplementary Table S7. Time-varying association between tumor-tissue pgRNA expression and postoperative outcomes.

Panel A. Time-varying coefficient Cox models

| Endpoint | HR at 12 months (95% CI)† | *P* | Time-varying coefficient, β | *P* for time interaction | Tumor size HR (95% CI) | *P* |
| --- | --- | --- | --- | --- | --- | --- |
| OS | 1.27 (1.01–1.61) | 0.045 | −0.367 | 0.018 | 1.20 (1.07–1.34) | 0.002 |
| RFS | 1.27 (1.05–1.54) | 0.016 | −0.336 | 0.010 | 1.18 (1.05–1.32) | 0.005 |

Panel B. Estimated time-specific HRs for tumor-tissue pgRNA

| Time after surgery | OS HR (95% CI) | RFS HR (95% CI) |
| --- | --- | --- |
| 6 months | 1.64 (1.08–2.50) | 1.60 (1.14–2.26) |
| 12 months | 1.27 (1.01–1.61) | 1.27 (1.05–1.54) |
| 18 months | 1.10 (0.94–1.28) | 1.11 (0.96–1.28) |
| 24 months | 0.99 (0.85–1.14) | 1.01 (0.87–1.16) |
| 36 months | 0.85 (0.69–1.04) | 0.88 (0.72–1.07) |
| 48 months | 0.76 (0.58–1.01) | 0.80 (0.62–1.03) |

Panel C. 24-month piecewise Cox sensitivity analysis

| Endpoint | Follow-up period | HR per 2-fold increase (95% CI) | *P* | *P* for early-vs-late interaction |
| --- | --- | --- | --- | --- |
| OS | 0–24 months | 1.21 (0.98–1.48) | 0.070 | 0.006 |
|  | >24 months | 0.85 (0.70–1.04) | 0.117 |  |
| RFS | 0–24 months | 1.19 (0.98–1.43) | 0.072 | 0.091 |
|  | >24 months | 0.92 (0.73–1.15) | 0.442 |  |

**Notes:** Tumor-tissue pgRNA was modeled as log₂-transformed relative expression; HRs therefore represent the change in hazard per two-fold increase. Time-varying Cox models were adjusted for tumor size and specified β(t) = β₁₂ + βtime × log(t/12). Time-specific HRs were derived from the same fitted model. The 24-month piecewise Cox model was performed as a sensitivity analysis. HRs <1 during later follow-up should not be interpreted as protective because their 95% CIs included 1.
